# Cerebral Amyloid Angiopathy-Related Inflammation (CAA-ri): Presentation at an Unusual Age

**DOI:** 10.7759/cureus.42454

**Published:** 2023-07-25

**Authors:** Diego Zúñiga, Gabriel Zúñiga, Sofía Hincapié, Erin Salazar

**Affiliations:** 1 Facultad de Ciencias Médicas, Universidad Católica de Santiago de Guayaquil, Guayaquil, ECU; 2 Neurology, Hospital Luis Vernaza, Guayaquil, ECU

**Keywords:** corticosteroids, white matter hyperintensity lesions, amyloid-beta, dementia, alzheimer’s disease, cerebral amyloid angiopathy-related inflammation, cerebral amyloid angiopathy

## Abstract

Cerebral amyloid angiopathy-related inflammation (CAA-ri) is a less common but aggressive manifestation of CAA caused by an autoimmune reaction to the amyloid-beta (Ab) deposits in affected vessels. Here, we report the case of a 96-year-old patient, with a history of Alzheimer's disease, who presented to our hospital due to a sudden onset of high-intensity holocranial headache followed by dysarthria, left hemiplegia, and gaze deviation to the right. MRI of the brain was performed, which revealed a heterogeneous hypointense signal on the right frontal T2 and fluid-attenuated inversion recovery (FLAIR) sequences, with an asymmetric hyperintensity surrounding the lesion compatible with perilesional vasogenic edema. Given the clinical radiographic findings, a diagnosis of CAA-ri was established and immediate treatment with intravenous corticosteroids was started, with a rapid clinical response and remarkable improvement in follow-up neuroimaging.

## Introduction

Cerebral amyloid angiopathy (CAA) is a cerebrovascular disease characterized by an abnormal buildup of amyloid-beta (Ab) in the leptomeninges and the smaller- to medium-sized blood vessels of the brain [1]. A less common manifestation of CAA is CAA-related inflammation (CAA-ri). Patients typically have a younger age compared to those with CAA, with an average age of 67 years [2]. The primary symptoms commonly observed in CAA-ri include headaches, seizures, focal neurological impairments, and a gradual to sudden decline in cognitive abilities [2-4]. A diagnosis can be established by evaluating the clinical presentation and radiological findings observed on MRI. The optimal treatment for CAA-ri has not yet been clearly defined; however, it relies on intensive immunosuppression, predominantly with steroids [5]. 

In this case report, we describe the clinical scenario of a patient who presented with sudden neurological impairment and cognitive alteration. Our patient’s age was 96 years, thus placing her at the extreme end of the spectrum in comparison with the average age of CAA-ri patients. Imaging and clinical context were used to make the diagnosis of CAA-ri. Furthermore, the positive clinical and radiologic response to treatment with corticosteroids was also supportive of the diagnosis.

## Case presentation

A 96-year-old female patient with a medical history of hypothyroidism and Alzheimer's disease (AD) treated with levothyroxine and memantine, respectively, presented to our hospital due to a sudden onset of high-intensity holocranial headache and dysarthria, followed by a generalized tonic-clonic seizure.

Upon admission, the patient was awake and disoriented in time and space. Her neurological examination revealed mild dysarthria, left-side hemiplegia with sphincter dysfunction, and gaze deviation to the right. The remainder of the physical examination was normal. Vital signs were within normal limits. The patient denied head trauma and any activity or stimuli that could have triggered her symptoms. She was not using anticoagulants and did not have any history of cancer.

**Figure 1:**
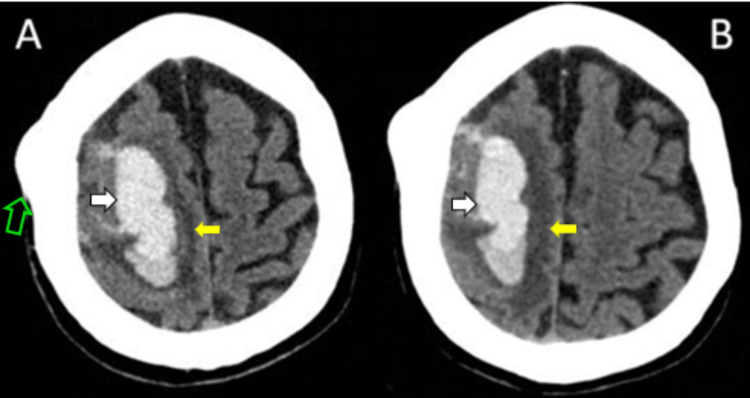
(A) Initial axial CT scan of the head showing right frontal hemorrhage (white arrows) with a surrounding hypodensity suggestive of a perihemorrhagic edema (yellow arrows). (B) After 48 hours, an increase in perilesional edema was noted. An osteoma in the right frontal bone is also seen (green arrow). CT: Computed tomography

A head CT was performed, which showed a right frontal hyperdensity consistent with a hemorrhage of approximately 20 ml in volume with a surrounding hypodensity suggestive of a perihemorrhagic edema (Figure 1A). Close monitoring and supportive care as well as other measures for intracerebral hemorrhage were considered for this patient. Phenytoin was also administered. After 48 hours, the patient presented an acute decline in sensorium, reaching a stupor, for which a new head CT was performed, showing an increase in the perilesional edema (Figure 1B).

**Figure 2:**
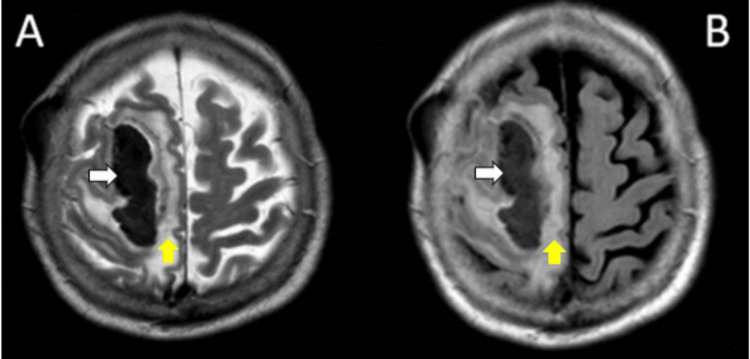
Axial T2W (A) and FLAIR (B) MRI sequences of the brain showing a hypointense signal (white arrows) on the right frontal lobe with perilesional vasogenic edema (yellow arrows). T2W: T2-weighted image; FLAIR: fluid attenuated inversion recovery; MRI: magnetic resonance imaging

Due to her medical history of AD, a diagnosis of CAA-ri was suspected. Subsequently, an MRI of the brain was performed, which showed a heterogeneous hypointense signal on the right frontal lobe T2 and fluid-attenuated inversion recovery (FLAIR) sequences, with an asymmetric hyperintensity surrounding the lesion compatible with perilesional vasogenic edema (Figure 2). Angio MRI showed a reduced diameter in the anterior and middle cerebral arteries attributed to atherosclerotic changes. 

Complete blood count and metabolic panel showed no abnormalities. The coagulation profile (including platelets, prothrombin time, international normalized ratio, partial thromboplastin time, and coagulation time) was within range. Thyroid hormones were normal and inflammatory markers were not elevated. In order to eliminate the possibility of a cancer-related cause, tumor markers (CA 125, CEA, CA 19-9) were ordered as well as CT and echo scans of the chest and abdomen, all of which were unremarkable. An electroencephalogram (EEG) showed slow focal activity in the right frontal lobe. A lumbar puncture was performed and cerebrospinal fluid (CSF) biochemical and cytological analysis showed elevated CSF protein level (0.75 g/L) with normal glucose and cell counts. Immunological and microbiologic CSF analysis was unremarkable. 

**Figure 3:**
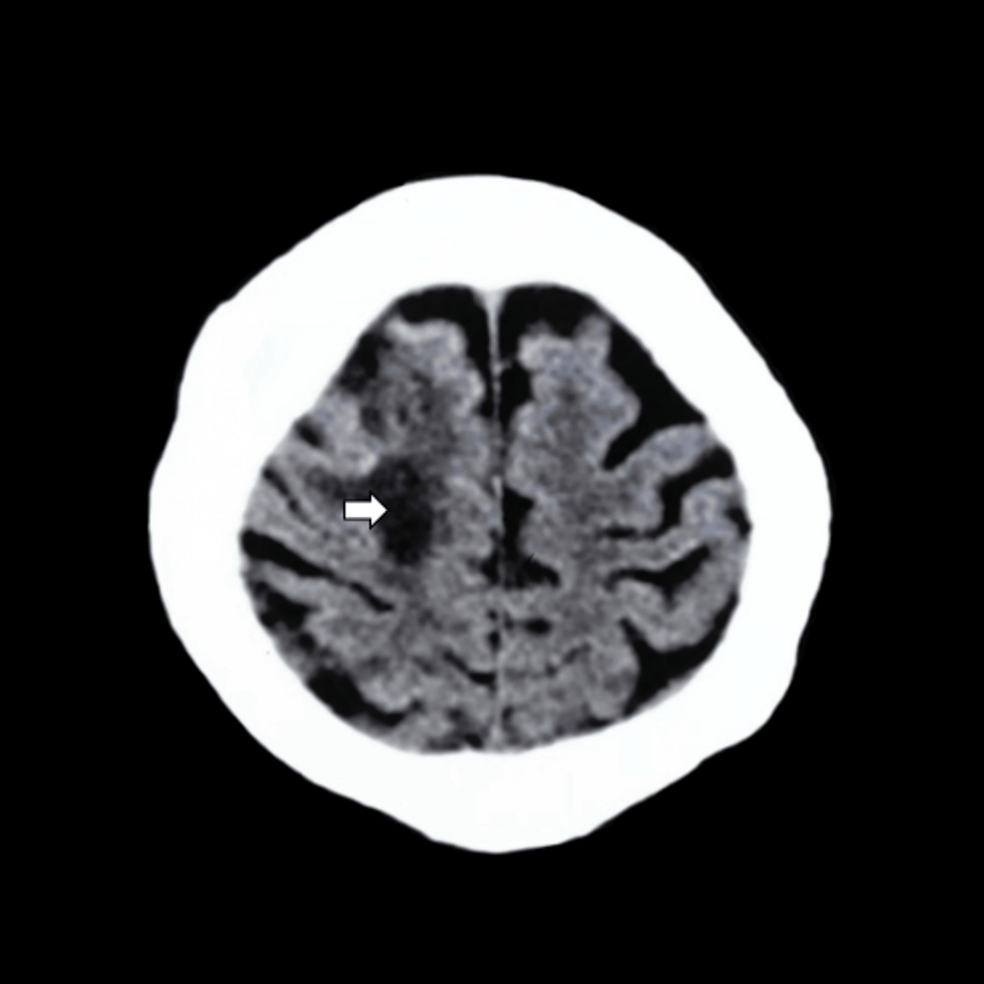
An axial CT scan of the head two months after therapy initiation showing a hypodense sequelae lesion (white arrow) with a marked reduction of the perilesional edema. CT: computed tomography

Treatment with intravenous corticosteroids was started, with a good response after 24 hours. The patient was awake and cooperative but still disoriented in time and space. The neurological assessment showed left hemiplegia, mild dysarthria, and resolution of the gaze deviation. She was discharged with severe left motor sequelae, mild dysarthria, and progressive tapering of steroids. A follow-up head CT at two months showed a hypodense sequelae lesion with a marked reduction of the perilesional edema (Figure 3). 

## Discussion

Cerebral amyloid angiopathy (CAA) is a cerebrovascular disease characterized by an accumulation of amyloid-beta (Ab) in the leptomeninges and the smaller to medium-sized blood vessels of the brain [1]. Spontaneous lobar intracerebral hemorrhages (ICH) are the most prevalent clinical manifestation, as well as smaller areas of bleeding such as cerebral microbleeds (CMBs) and cortical superficial siderosis (cSS) [6]. CAA predominantly occurs as a sporadic condition in older individuals or as a hereditary familial form [7]. The presence of CAA is closely tied to age, as the prevalence of moderate to severe CAA rises significantly with increasing age [8]. In cases of AD, CAA is frequently observed, with an occurrence rate of approximately 80-90% [9-12]. CAA is not significantly associated with common risk factors for cerebrovascular conditions like hypertension, diabetes mellitus, and hyperlipidemia [9].

A less common manifestation of CAA is CAA-related inflammation (CAA-ri). The primary symptoms commonly observed in CAA-ri include headaches, seizures, focal neurological impairments, and a gradual to sudden decline in cognitive abilities [2-4]. It is believed that CAA-ri occurs as a result of an inflammatory reaction to Ab within the blood vessel walls. Individuals diagnosed with CAA-ri typically have a younger age compared to those with CAA, with an average age of 67 years [2]. In contrast, the age of our patient was 96. The clinical progression of CAA-ri can vary. However, it is generally observed that immunosuppressive therapy tends to elicit a favorable response in most cases.

In the past, it was common clinical practice to conduct a brain biopsy in all suspected cases of CAA-ri prior to initiating immunosuppression. However, now a diagnosis can be established by evaluating the clinical presentation and radiological findings observed on MRI. Nevertheless, a definitive diagnosis of CAA-ri still requires a cerebral biopsy.

MRI findings typically include the presence of asymmetric hyperintense lesions that appear as confluent or patchy areas on T2-weighted or FLAIR sequences with or without edema, multiple cortical and/or subcortical microhemorrhages, and post-contrast enhancement of the leptomeninges [13]. Sometimes, these findings may resemble those of malignant lesions, adding to the diagnostic challenge.

The understanding that MRI findings can accurately indicate CAA-ri when considered within the appropriate clinical context led Chung et al. to the development of diagnostic criteria [2]. 

In 2016, Auriel et al. [3] made revisions to the criteria established by Chung et al. These updates included the definition of a specific pattern of white matter hyperintensities (WMH) to differentiate between probable and possible CAA-ri (Table 1). Additionally, they suggested cSS as an indicator of hemorrhage [3]. 

**Table 1:** Criteria for the Diagnosis of CAA-ri CAA-ri; cerebral amyloid angiopathy-related inflammation; ICH, intracerebral hemorrhage; MRI, magnetic resonance imaging; WMH, white matter hyperintensity

Probable CAA-ri	Possible CAA-ri
Age ≥40 years	Age ≥40 years
Presence of ≥1 of the following clinical features: headache, decrease in consciousness, behavioral change, or focal neurological signs and seizures; the presentation is not directly attributable to an acute ICH	Presence of ≥1 of the following clinical features: headache, decrease in consciousness, behavioral change, or focal neurological signs and seizures; the presentation is not directly attributable to an acute ICH
MRI shows unifocal or multifocal WMH lesions (corticosubcortical or deep) that are asymmetric and extend to the immediately subcortical white matter; the asymmetry is not due to past ICH	MRI shows WMH lesions that extend to the immediately subcortical white matter
Presence of ≥1 of the following corticosubcortical hemorrhagic lesions: cerebral macrobleed, cerebral microbleed, or cortical superficial siderosis	Presence of ≥1 of the following corticosubcortical hemorrhagic lesions: cerebral macrobleed, cerebral microbleed, or cortical superficial siderosis
Absence of neoplastic, infectious, or other cause	Absence of neoplastic, infectious, or other cause

Our patient presented with an acute onset of headache, alteration in mental status, focal neurological signs, and a seizure. This, combined with radiological evidence on MRI that confirmed a right frontal lobar hemorrhage associated with an asymmetric vasogenic edema and cortical superficial hemosiderosis, fall into the probable category without histopathological confirmation as described by Auriel (2016).

CAA-ri exhibits similar pathological features to CAA, which involves the deposition of Ab in the vessels of the cortex or leptomeninges, as confirmed by positive Congo red staining. In addition to Ab deposition, CAA-ri also demonstrates notable perivascular or trans mural inflammatory infiltration [14]. CAA-ri consists of two subtypes: inflammatory cerebral amyloid angiopathy and amyloid-beta (Ab)-related angiitis [15]. 

CAA-ri often presents with elevated inflammatory markers. Our patient did not have a marked elevation of inflammatory markers. To help rule out infectious diseases, a cerebrospinal fluid (CSF) examination is necessary. The CSF analysis commonly reveals abnormalities such as lymphocytic pleocytosis and/or elevated protein levels [16]. Electroencephalography (EEG) typically reveals non-specific findings such as focal or generalized slowing and epileptiform changes [2]. Focal slowing in the right frontal region was found in the EEG of our patient.

Additional diagnostic evidence for CAA-ri can be obtained through the identification of genotypes, anti-Ab autoantibodies, and amyloid PET scans [17]. However, it is worth noting that antibody titer determination kits are currently not commercially accessible, and amyloid PET scans are not widely accessible either.

The primary conditions to consider as potential alternative diagnoses include posterior reversible encephalopathy syndrome (PRES) [17], viral or autoimmune encephalitis, cerebral venous thrombosis, neurosarcoidosis, acute disseminated encephalomyelitis (ADEM) [18], and malignant processes like primary CNS lymphoma [19], carcinomatous meningitis, and gliomatosis cerebri.

The optimal treatment for CAA-ri has not yet been clearly defined. The treatment of CAA-ri relies on intensive immunosuppression, predominantly with steroids [5]. Rapid clinical and radiological responses have been observed with the use of steroids alone. A typical treatment course involves a high-dose intravenous steroid pulse followed by a prolonged steroid taper (at least six months). However, approximately 20-30% of patients either do not respond well to steroid treatment or experience recurrent symptoms, for whom mycophenolate and azathioprine may be considered [5]. 

A short course of steroids was sufficient to achieve clinical improvement in our patient, without relapses for two months. Nevertheless, relapses have been reported after treatment completion, thus the optimal duration of treatment still needs to be determined.

## Conclusions

In conclusion, CAA and its inflammatory manifestation, CAA-ri, are complex conditions that require careful evaluation and diagnosis. The diagnosis of CAA-ri can be challenging, but clinical symptoms, radiological findings on MRI, and other diagnostic tools such as CSF examination and EEG can aid in the evaluation. There are specific criteria proposed by researchers like Chung et al. and Auriel et al. to assist in the diagnosis. In the case presented, the patient exhibited characteristic imaging findings and clinical response to a short course of steroids, supporting the diagnosis. Prompt diagnosis is crucial for favorable treatment outcomes, as delayed diagnosis may result in partial and less satisfactory recovery. Recent findings have shown elevated anti-Ab antibody titers in CSF in patients with CAA-ri, which decreased after corticosteroid therapy, suggesting that anti-Ab antibodies could play a future role in monitoring therapy response and noninvasive diagnosis. Further studies are still needed to continue to improve our understanding and management of this condition.
